# The Impact of Stimulation Parameters on Cardiovascular Outcomes in Chronic Stroke Patients Following Transcranial Direct Current Stimulation—A Pilot Controlled, Randomized, Double-Blind Crossover Trial

**DOI:** 10.3390/biomedicines12091998

**Published:** 2024-09-02

**Authors:** Fernanda Ishida Corrêa, Laura Uehara, Michele Lacerda de Andrade, Gustavo Oliveira da Silva, Katia De Angelis, Ariane Viana, Catarina Novaes Souza Bertani, João Carlos Ferrari Corrêa, Felipe Fregni

**Affiliations:** 1 Doctoral and Master Program in Science of Rehabilitation, Nove de Julho University, Rua Vergueiro 235/249, São Paulo 01504-001, Brazil; lor.uehara@gmail.com (L.U.); michele.la.andrade@gmail.com (M.L.d.A.); gustavo.pnt@hotmail.com (G.O.d.S.); prof.kangelis@uni9.pro.br (K.D.A.); a.viana@uni9.pro.br (A.V.); ishida.correa@gmail.com (C.N.S.B.); jcorrea@uninove.br (J.C.F.C.); 2 Neuromodulation Center and Center for Clinical Research Learning, Spaulding Rehabilitation Hospital and Massachusetts General Hospital, Harvard Medical School, Boston, MA 02129, USA; fregni.felipe@mgh.harvard.edu

**Keywords:** autonomic modulation, functional capacity, heart rate variability, stroke, transcranial direct current stimulation

## Abstract

Background: Stroke survivors often experience autonomic nervous system (ANS) dysfunction. While Transcranial Direct Current Stimulation (tDCS) has been shown to modulate the ANS when applied to the left hemisphere, its effects on the right hemisphere remain unexplored. Objective: We aimed to compare the effects of tDCS applied to both the injured and the contralateral hemispheres on heart rate variability (HRV) and functional capacity in individuals post-stroke. Methods: Twenty individuals with cerebral hemisphere lesions (ten with right-hemisphere lesions and ten with left-hemisphere lesions) were randomized into four groups: anodal and sham tDCS on the left temporal cortex (T3) and anodal and sham tDCS on the right temporal cortex (T4). HRV was assessed before the intervention, after the six-minute walk test (6MWT), and following tDCS. HRV data were categorized into frequency ranges: low frequency (LF), high frequency (HF), and sympathovagal balance. The 6MWT (meters) was conducted both pre- and post-tDCS. Results: In individuals with right-hemisphere lesions, a higher global LF value was observed (right side: 71.4 ± 16.8 nu vs. left side: 65.7 ± 17.3 nu; *p* = 0.008), as well as lower values of the HF component (right side: 29.5 ± 18.9 nu vs. left side: 34.0 ± 17.4 nu; *p* = 0.047), consequently exhibiting higher global values of the low/high-frequency ratio (right side: 3.9 ± 2.8 vs. left side: 2.9 ± 2.4). Regarding the stimulation site, tDCS over T3 led to a lower overall value of the low/high-frequency ratio (left hemisphere: 3.0 ± 2.2 vs. right hemisphere: 3.7 ± 2.9; *p* = 0.040) regardless of the lesion location. A significant increase in the distance covered in the 6MWT was observed for individuals with lesions in both hemispheres after tDCS at T3. Conclusions: Participants with right-hemisphere lesions exhibited superior global sympathetic autonomic nervous system activity. When the tDCS was applied on the left hemisphere, it maintained lower sympathovagal balance values and improved functional capacity regardless of the hemisphere affected by the stroke.

## 1. Introduction

Stroke survivors may experience cardiovascular dysfunction due to changes in the autonomic nervous system (ANS), regardless of the affected hemisphere [1]. However, when the involvement occurs in the right hemisphere, there is a predominance of sympathetic over parasympathetic tone [2], which can provoke a stroke of greater severity and greater risk of early death [3,4,5]. Cha et al. [6] also reported that autonomic dysfunction caused by stroke, in addition to increasing mortality, impairs functional results.

These autonomic changes frequently occur in the acute phase of stroke [7]; however, they can be maintained in the chronic phase, as demonstrated by Constantinescu et al. [8], Dütshc et al. [9], Damkjaer et al. [10], and Heinz et al. [11], who observed low parasympathetic activity and a prevalence of sympathetic activity in individuals with chronic stroke sequelae.

Transcranial Direct Current Stimulation (tDCS) has been investigated as an intervention to modulate the autonomic nervous system when applied to the left temporal cortex (T3) [12,13], with promising results in aerobic performance, reducing the perception of effort, decreasing the performance of the sympathetic system, and improving the post-exercise recovery of athletes. In hemiparetic individuals with stroke, tDCS over T3 led to an increase in parasympathetic tone [11].

Given these findings, this study aimed to evaluate the sympathetic vagal balance of individuals who suffered strokes in the right or left hemispheres and to verify the effects of tDCS on heart rate variability (HRV) and functional capacity (FC) when applied to the injured or non-injured hemispheres of these individuals.

## 2. Materials and Methods

### 2.1. Design

**Figure 1:**
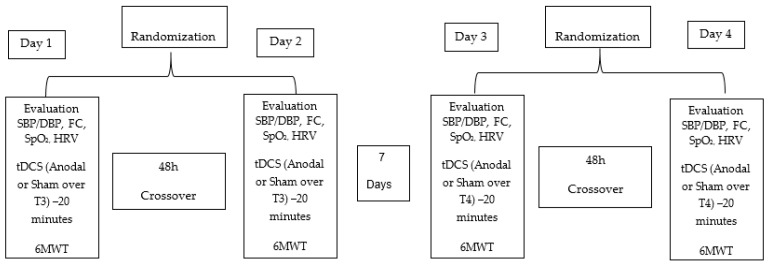
Study timeline—SBP (systolic blood pressure), DBP (diastolic blood pressure), HR (heart rate), SpO_2_ (oxygen saturation %), HRV (heart rate variability), tDCS (Transcranial Direct Current Stimulation), T3 (left temporal cortex), T4 (right temporal cortex), 6MWT (six-minute walk test).

This study is a pilot double-blind, crossover, randomized, sham-controlled clinical trial. The study design timeline is shown in Figure 1.

The participants were randomized to receive four interventions with tDCS: 1—anodal tDCS (atDCS) over T3 and the cathode over the contralateral supraorbital region; 2—sham tDCS (stDCS) over T3 and the cathode over the contralateral supraorbital region; 3—atDCS over the right temporal cortex (T4) and the cathode over the contralateral supraorbital region; and 4—stDCS over T4 and the cathode over the contralateral supraorbital region.

Each intervention was performed once, with a minimum interval of 48 h and a maximum of one week between them and always at the same period of the day.

### 2.2. Ethical Aspects

The study protocol followed the standards established by the Consolidated Standards of Reporting Trials (CONSORT), following the principles of the Declaration of Helsinki and the standards for research with human beings formulated by the National Health Council of Brazil, created in October 1996. It received approval from the Ethics Committee for Research on Human Beings of the local Research Ethics Committee (protocol n° 738 356/2014) and was registered as a clinical trial (NCT 02398344).

### 2.3. Eligibility Criteria

Hemiparetic participants resulting from a stroke in the left or right hemisphere, confirmed by a doctor and complementary exams more than six months ago in patients aged between 49 and 71 years old and scoring between levels 04 and 06 on the Functional Mobility Scale (FMS) [14], were included in this study.

Participants with cognitive impairment (scores below 24) on the Mini-Mental State Examination (MMSE) [15]; who had visual impairment that could interfere with the exams; who made use of pacemakers; and who had contraindications to the use of tDCS, such as metallic implants in the head or close to the stimulation site, a history of recent and uncontrolled epilepsy, and scalp injuries [16], were not included. The eligible participants signed the informed consent form, and their information was kept anonymous.

### 2.4. Sample Recruitment and Selection

The sample consisted of 20 participants of both genders, recruited from the waiting lists of the Nove de Julho University physiotherapy clinic in São Paulo (Brazil) through telephone contact and indications from health professionals.

### 2.5. Transcranial Direct Current Stimulation

The tDCS was performed using the Tct Research 1 CH tDCS Simulator model 101^®^ device, using two 5 × 7 cm^2^ surface sponge electrodes (non-metallic) moistened in 0.9% saline solution for better conduction of the electric current and applied to the scalp, fixed by two hypoallergenic rubber bands with the aid of two plastic pins, with a current of 2 mA for 20 min.

The anodal electrode was positioned on the T3 or T4 coordinates (depending on randomization), and the cathode was on the contralateral supraorbital region. The positioning followed the measurements of the 10/20 electroencephalogram (EEG) system [17]. In the sham stimulation, all the electrodes were placed similarly to the anodal tDCS procedure, but the device remained on for only 30 s.

### 2.6. Assessments

All participants were instructed to have light meals on the intervention days; abstain from consuming caffeine, alcohol, or tobacco; and avoid moderate or excessive exertion on the day before and the day of the test.

To characterize the sample, we collected personal and clinical data, such as full names, dates of birth, ages, topographic diagnoses, classifications (hemorrhagic or ischemic) of the strokes, time of injury, and medications used.

### 2.7. Heart Rate Variability

The HRV assessment was carried out in the following periods: pre-intervention (baseline [HRV-1]) for 10 min; after the six-minute walk test (6MWT) for 2 min (HRV-2), and after tDCS for 2 min (HRV-3). For this assessment, the participant remained seated and was instructed not to move or talk so as not to interfere with the capture of the electrical signal.

The device used for the HRV data collection was a heart rate monitor (Polar^®^ RS800 CX). The HRV data were analyzed using the HRV analysis program in the Fast Fourier Transform (FFT) format, expressed in standard units (nu). After normalization, the relative powers were obtained in the respective pre-defined frequency bands: low frequency (LF) from 0.04 to 0.15 Hz, high frequency (HF) from 0.15 to 0.4 Hz, and sympathovagal balance (LF/HF).

### 2.8. Functional Capacity Assessment

Initially, subjective perceptions of respiratory effort and the lower limbs were measured using the modified Borg scale [18], systolic blood pressure (SBP), and diastolic blood pressure (DBP) as well as an aneroid sphygmomanometer (Premium^®^), oxygen saturation (SpO_2_) using a portable oximeter (More Fitness^®^), and heart rate (HR). Then, functional capacity (FC) was assessed by the 6MWT, in meters, at two moments, pre- (6MWT-1) and post-tDCS (6MWT-2), following the standards established by the American Thoracic Society [19]. The distances in the test at baseline and after tDCS, performed by the participant, were compared and expressed as mean and standard deviation.

### 2.9. Sample Size

This is a pilot study design, arbitrarily defined as 20 participants (10 in each group). A post hoc power analysis of the observed effect sizes for the primary outcomes, specifically differences in heart rate variability (HRV) and functional capacity (measured by the six-minute walk test) between the intervention groups, showed a size of effect of 0.25. Given this effect size, the alpha level of 0.05, and the total sample size of 20 participants, the post hoc power analysis revealed that this study had a power of approximately 0.72 to detect significant differences in the primary outcomes. The sample size was computed considering the most complex condition: the interaction between the hemisphere of injury and the type of stimulation. This interaction was expected to yield the smallest effect size, thereby driving our post hoc sample size determination.

### 2.10. Randomization

The order of the stimulations was randomized using brown envelopes to ensure confidentiality. The investigators responsible for the evaluation procedures and the participants were blinded to the treatment. Only the investigator responsible for coding the electrical stimulus was not blinded; however, he was not involved in the evaluation and revaluation after the procedure.

### 2.11. Statistical Analysis

The data were analyzed using the SPSS version 20 statistical program, according to the nature of the distribution of the variables, after checking the normality using the Kolmogorov–Smirnov test; the measures of central tendency and dispersion were the mean and standard deviations for the parametric data, and the median and interquartile range were for non-parametric data.

Generalized Estimated Equations (GEEs) were used to compare the effects of the experimental conditions on HRV, followed by a post hoc analysis of pairwise comparisons using the Bonferroni correction for multiple comparisons. A value of *p* < 0.05 was considered significant.

The mean and standard deviations for age and the HR (bpm), BP (mmHg), and SpO_2_ (%) baselines were used to characterize the samples; injury time by the median and interquartile ranges, as well as frequency and percentage, were used to determine the injured hemispheres (right and left), stroke subtypes (ischemic and hemorrhagic), and drug use.

The HRV power spectra in the frequency domain were quantified by measuring the areas of LF, HF, and the LF/HF through the interaction of sympathetic and parasympathetic activities.

## 3. Results

This study included 20 hemiparetic individuals due to chronic stroke, 10 with vascular lesions in the right hemisphere and 10 with vascular lesions in the left hemisphere (Supplementary Material Figure S1—Consort flowchart).

The clinical characteristics of the individuals are in Table 1:

In Table 1, it is possible to observe that most participants had ischemic strokes and more than half were using beta-blockers. Participants with lesions in the right and left hemispheres were homogeneous concerning HR, SBP, DBP, SpO_2_, and HRV in the frequency domain, represented by the LF and HF spectral bands and the LF/HF ratio at baseline.

Participants with lesions in both the right and left hemispheres showed a predominance of sympathetic modulation observed by the LF band (n.u) compared to the HF parasympathetic activity (n.u) confirmed by LF/HF > 1.

**Figure 2:**
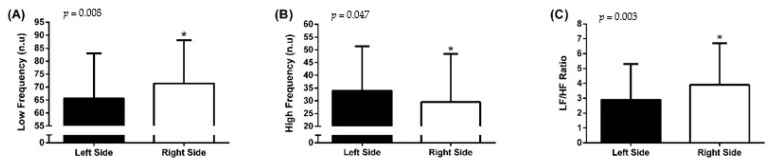
Comparison of overall low frequency (**A**), high frequency (**B**), and sympathovagal balance (LF/HF) (**C**) results on the left and right injured hemispheres. Normalized unit (n.u.). * *p* ≤ 0.05. Data expressed as overall mean ± standard deviation.

Figure 2 shows the results of the LF, HF, and LF/HF ratio between subjects with injury on the left and right sides.

The results indicated superior modulation of the LF component (71.4 ± 16.8 n.u., right side vs. 65.7 ± 17.3 n.u., left side; *p* = 0.008), lower modulation of the HF component (29.5 n.u. ± 18.9 n.u., right side vs. 34.0 ± 17.4 n.u., left side; *p* = 0.047), and superior modulation of the LF/HF ratio (3.9 ± 2.8, right side vs. 2.9 ± 2.4, left side; *p* = 0.003) in individuals with lesions in the right hemisphere compared with participants with lesions in the left hemisphere, regardless of the location of the stimulation.

**Figure 3:**
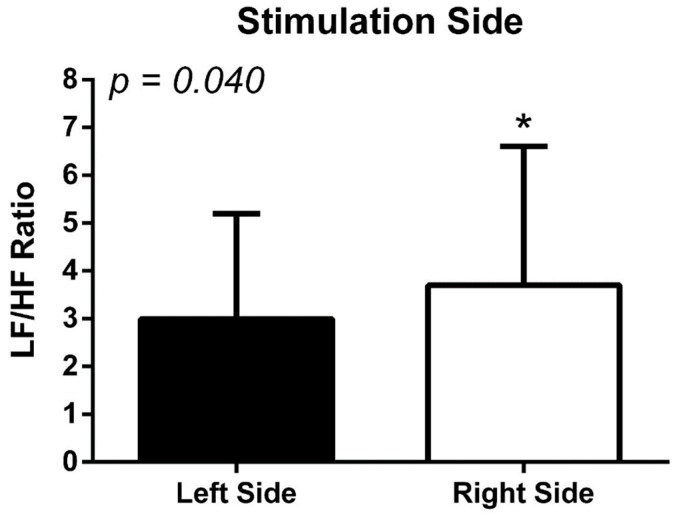
Ratio of sympathovagal balance (LF/HF) in relation to the side (left or right hemisphere) in which tDCS was applied, regardless of the injured hemisphere. * *p* ≤ 0.05.

Figure 3 shows the higher modulation of the LF/HF ratio between the stimulation on the left and right sides.

Regarding the stimulation site, a lower value of the LF/HF ratio was observed for stimulation performed on the left side compared with the right side (3.0 ± 2.2, left side vs. 3.7 ± 2.9, right side; *p* = 0.040), regardless of the side with the injury.

The absolute values (ms^2^) of LF, HF, and LF/HF of participants with right- and left-side lesions after receiving active and sham tDCS at T3 and T4 in the pre-and post-intervention conditions for active and sham stimulation have been entered in Table 2 (Supplementary Material Table S1).

The absolute values reinforce that individuals with lesions in both the right and left hemispheres presented a predominance of sympathetic modulation observed by the LF band (absolute values) compared to the parasympathetic HF action (absolute values), confirmed by the sympathovagal balance (LF/HF) > 1. The results after stimulation are similar to the results in normalized units.

Table 2 describes the results of the functional capacity of individuals with lesions on the left and right, pre- and post-tDCS, active and sham, with stimulations at T3 and T4.

There were significant increases (*p* = 0.00) in the walking distance, of 25.6 m for individuals with lesions in the right hemisphere and 31 m for individuals with lesions in the left hemisphere (*p* = 0.00), after receiving atDCS over T3.

When receiving the atDCS on T4, there was a non-significant increase of 32 m in the distance walked for participants with right-hemisphere lesions (*p* = 0.28) and 11.5 m for participants with left-hemisphere lesions (*p* = 0.50).

## 4. Discussion

This study aimed to compare the sympathovagal balance between individuals who suffered strokes in the right and left hemispheres and verify the effects of tDCS on HRV and FC when applied to the injured or non-injured hemispheres of these individuals.

### 4.1. Basal Condition

Our results showed that 100% of the participants, regardless of the injured hemisphere, showed superior sympathetic activity in the baseline condition, observed by a higher LF band index concerning the HF index and confirmed by an LF/HF greater than 2. These results corroborate Dorrance and Fink [1] and Yperzeele et al. [20], who reported this sympathetic predominance over the parasympathetic after stroke during the acute phase, and the studies by Heinz et al. [11], Grilletti et al. [21], and Lees et al. [22] of individuals already in the chronic phase of stroke.

Therefore, improving vagal sympathetic balance in these individuals is extremely important so that they do not increase the chances of having other cardiovascular episodes such as another stroke, heart attack, or even death [3,19].

### 4.2. After Stimulation

Regardless of the period evaluated, the participants with right-hemisphere lesions showed superior sympathetic modulation to those with left-hemisphere lesions. This result is reinforced by the sympathovagal balance results, which show that the participants with right-hemisphere lesions presented higher LF/HF ratio values than those with left-hemisphere lesions, regardless of time. Our results also indicate that stimulation performed on the right side, regardless of the side lesion, leads to superior increases in LF/HF. Reinforcing this finding, Contantinescu et al. [8], when evaluating the impact of middle cerebral artery (MCA) ischemic stroke on cardiac autonomic function during sympathetic and parasympathetic activation tests, observed that patients who had right MCA ischemic stroke showed decreased heart vagal modulation compared with healthy controls and patients who had left MCA ischemic stroke in the resting state and during autonomic activation tests. These results have been reinforced by human studies carried out by Hilz et al. [23] and Oppenheimer et al. [24], who examined the effect of stimulation or inactivation of both hemispheres, suggesting a crucial role of the right hemisphere in establishing sympathetic tone and of the left hemisphere in determining parasympathetic tone.

Therefore, we highlight our findings as essential because they show that the left hemisphere is the safest place to stimulate in modulating the autonomic nervous system, regardless of the lesion side. After all, even though the sympathovagal balance of the participants in this study did not reach the ideal values between 1.5 and 2.02 [1] after tDCS in the left hemisphere, the sympathovagal balance, which tends to be increased in these individuals, did not increase either, unlike in the results when the stimulus occurred in the right hemisphere. These results corroborate Montenegro et al. [12] and Okano et al. [13], who found an increase in HF and a decrease in LF and LF/HF in athletes after receiving stimulation in the left temporal cortex, and Contantinescu et al. [8], who observed that the parasympathetic activation test did not change the sympathovagal balance in individuals who had suffered strokes.

Another outcome evaluated in our study was functional capacity, measured by the distance covered in meters. A significant increase in distance traveled was observed for participants with left- and right-hemisphere lesions after receiving anodal tDCS over the T3 cortex. Roncato et al. [25] reported that parasympathetic action, which is the responsibility of the T3 area, is essential for optimizing functional capacity, as during physical effort, parasympathetic action helps to reduce the sensation of stress and psychophysiological fatigue, favoring the maintenance of performance. Similar results were reported by Okano et al. [13], who observed improved performance in healthy cyclists after receiving atDCS on the T3 before exercise, and also by Heinz et al. [11], who observed an increase in the distance covered on a treadmill by individuals with chronic stroke after using atDCS on T3.

Ulrich-Lai and Herman [26] investigated the effects of autonomic dysfunction on functional outcomes in patients with acute stroke and during their recovery. They observed that the severity of autonomic dysfunction influences the functional prognosis in patients with acute stroke; i.e., autonomic dysfunction above the moderate level was associated with poorer functional recovery than mild autonomic dysfunction. According to those authors [26], autonomic dysfunction can lead to impaired functional results because the dizziness and orthostatic hypotension characteristic of this dysfunction can reduce the frequency and intensity of rehabilitation; another factor would be that dysregulation of the sympathetic and parasympathetic nervous systems can lead to an insufficient blood supply to injured brain tissue, and finally, the autonomic nervous system is vital for regulating stress and maintaining homeostasis in response to the brain’s perception of stressors such as acute and chronic stroke.

### 4.3. Effects of tDCS on the Autonomic Nervous System

A previous systematic review [27] highlighted that many non-invasive brain stimulation studies have not demonstrated significant effects on the ANS. This result is often attributed to the stimulation parameters and the studied populations. Specifically, the review noted that a single session of tDCS does not typically induce significant effects on the ANSs in healthy individuals. However, a recent study [28] involving tDCS in individuals with spinal cord injuries—a group characterized by an unbalanced ANS—showed significant results, suggesting that the effectiveness of tDCS may also depend on the baseline ANS so that tDCS would be applicable to restore normality to this system.

In this context, our study, which targeted stroke survivors, may have induced significant effects due to multiple stimulation sessions and the specific characteristics of our population. Our findings indicate that these individuals exhibited an increased sympathetic tone at the beginning of this study, which probably enabled the modulation of the ANS by tDCS, as this method of cortical stimulation would trigger compensatory mechanisms to normalize the parasympathetic tone.

The exact mechanisms through which cortical stimulation affects the ANS remain unclear. It is hypothesized that cortical stimulation may exert a top-down influence on central autonomic networks, potentially modulating ANS centers in the brain via noradrenergic and serotonergic nuclei in the brainstem. This suggests a complex interplay between the site of stimulation and the broader neural network that governs autonomic functions, warranting further investigation into the specific pathways and mechanisms involved in animal studies and neuroimaging.

Another aspect that merits exploration is the connection between functional improvement and homeostasis, notably through astrocyte measurements. Additionally, these studies [29,30] indicate that astrocytes, during both the post-acute and chronic phases, seem to facilitate and inhibit neural plasticity processes, which are fundamental to functional recovery. Therefore, modulating astrocyte function by tDCS at this later stage seems an interesting approach to improve long-term outcomes for stroke survivors, as astrocytes control many functional aspects of the Central Nervous System (CNS) in health and disease, including maintaining CNS homeostasis, managing and supporting neurons, recycling neurotransmitters, controlling blood flow and induction, functional control, pruning of neuronal synapses, generating new neurons, sprouting axons, and controlling synapse number and function. Some studies, including a pilot study by Callai et al. [31], have demonstrated that tDCS can enhance neural plasticity and accelerate functional recovery by modulating astrocyte activation. For instance, the study by Callai et al. showed that a single session of tDCS could alter behavioral and neurochemical parameters, including astrocytic activation in rats. Similarly, Zhang et al. [32] found that tDCS significantly inhibited astrocyte activation under the pathological condition of ischemic stroke, and finally, Monai et al. [33] reported that tDCS could regulate the plasticity of astrocytes and cortex.

### 4.4. Assessment Parameters

To analyze our data, we chose the linear method in the frequency domain (spectral analysis) of the LF and AF components used to quantify, respectively, the modulation of the sympathetic and parasympathetic branches of the autonomic nervous system and the LF/HF ratio, as this method has been widely used since its introduction in the 1960s. Unlike the time domain, this technique decomposes the total variability of the signal into specific components that operate in different frequency bands, allowing identification [34,35].

While the LF component is typically associated with both sympathetic and parasympathetic sources, some studies suggest that the normalized value of the LF component may serve as a measure of sympathetic efferent activity [36,37]. Consequently, LF remains a commonly used metric in HRV analysis, as highlighted in a review study on the subject [38], because of its historical significance in understanding autonomic function. Our choice to include LF power was motivated by its established use in similar studies and its relevance to our research objectives. However, we recognize the limitations inherent in interpreting LF power in isolation and the potential for confounding factors in assessing sympathetic dominance.

## 5. Limitations and Conclusions

We emphasize that this is the first study that has compared the effects of tDCS on HRV in people with chronic strokes in the left and right hemispheres and the impact of this therapy on the injured hemisphere and contralateral to the injury. These findings contribute to the use of tDCS as a modulator of the autonomic nervous system safely and efficiently, thus being a resource to prevent new episodes of stroke and even prevent deaths. A limitation of this study is that we did not have imaging tests or neurophysiological tests to prove the sizes of the injured areas of the participants in this study. Another limitation is that although our sample size calculation mainly focused on the interaction between the hemisphere of injury and the type of stimulation, we acknowledge that the stimulation site, the injured versus uninjured hemisphere, was not precisely accounted for in the initial computation. This approach was established from preliminary data indicating that the variance associated with the stimulation site was less significant relative to the hemisphere of injury or type of stimulation. However, we recognize that this simplification may not fully account for the complexity of our study design.

## Figures and Tables

**Table 1:** Demographic clinical characteristics (n = 20).

Characteristic	Right Injury (N = 10)	Left Injury (N = 10)	*p*
Age (years) ^1^	60.3 ± 10.8	60.2 ± 9.6	0.05
Gender (F/M)	10 (3/7)	10 (5/5)	
Ischemic stroke ^2^	7 (70%)	6 (60%)	
Hemorrhagic stroke ^2^	3 (30%)	4 (40%)	
HR (bpm) ^1^	86.6 ± 15.85	81.9 ± 19.85	0.57
SBP (mmHg) ^1^	125 ± 22.73	126 ± 15.05	0.90
DBP (mmHg) ^1^	81 ± 15.95	79 ± 7.38	0.72
SpO_2_ (%) ^1^	95 ± 1.88	95.1 ± 1.97	0.12
Injury time (months) ^3^	67.5 (30.7–96.5)	40.0 (20.7–66.7)	
B-blocker medication ^2^	4 (40%)	9 (90%)	
ACE inhibitor medication ^2^	3 (30%)	1 (10%)	
No medication ^2^	3 (30%)	0	
HRV1			
LF (n.u.) ^1^	69.42 ± 13.48	65.7 ± 15.33	0.57
HF (n.u.) ^1^	30.58 ± 13.48	34.30 ± 15.33	0.57
LF/HF ^1^	2.60 ± 1.71	2.19 ± 1.71	0.62
6MWT (m) ^3^	227.7 (125.2–292.5)	333.5 (205–455)	

Data are presented as ^1^ mean ± SD; ^2^ frequency and percentage (%); and ^3^ median (interquartile range). F/M: female/male; HR: heart rate, bpm: beats per minute; SBP: systolic blood pressure; DBP: diastolic blood pressure; mmHg: millimeters of mercury; SpO_2_: oxygen saturation; ACE: angiotensin-converting enzyme; HRV: heart rate variability; LF: low frequency; HF: high frequency; n.u: normalized unit; LF/HF: sympathovagal balance; 6MWT: six-minute walk test. A significant level of *p* < 0.05 was set for all statistical analyses (independent-sample *t*-test).

**Table 2:** Results of the 6MWT pre- and post-treatment with tDCS applied on the injured and non-injured hemispheres of participants with injuries to the right and left hemispheres.

	Left injury (n = 10)					
Variables	Active tDCS on T3			Sham tDCS on T3		
	Pre-treatment	Post-treatment	*p*-value	Pre-treatment	Post-treatment	*p*-value
6MWT (m)	333.5 (205–455)	364.5 (259–481) *	0.00	316.5 (227.5–470.2)	326 (228–445.2)	0.83
	Active tDCS on T4			Sham tDCS on T4		
6MWT (m)	343.5 (227.5–81.7)	355 (261–476)	0.50	338.5 (223.5–498.7)	355 (261–476)	0.87
	Right injury (n = 10)					
Variables	Active tDCS on T3			Sham tDCS on T3		
6MWT (m)	227.7 (125.2–292.5)	253.3 (129.2–322.5) *	0.00	242 (142.5–328)	242 (142.5–328)	0.08
	Active tDCS on T4			Sham tDCS on T4		
6MWT (m)	221 (135.5–312.2)	253 (124–319)	0.28	229 (137.2–351.2)	238.5 (134.2–331)	0.95

Legend: Data expressed as median and interquartile ranges; 6MWT: six-minute walk test; m: meters; tDCS: Transcranial Direct Current Stimulation; T3: left temporal cortex; T4: right temporal cortex; * *p* < 0.05.

## Data Availability

The data presented in this study are available on request from the corresponding authors.

## Supplementary Materials

The following supporting information can be downloaded at: https://www.mdpi.com/article/10.3390/biomedicines12091998/s1, Figure S1: Study flowchart. Table S1. The absolute values (ms2) of LF, HF and LF/HF of participants with right and left lesions after receiving active and sham tDCS on T3 and T4, in the pre and post intervention conditions for active and sham stimulation.
