# Supplementary material for: The Impact of Stimulation Parameters on Cardiovascular Outcomes in Chronic Stroke Patients Following Transcranial Direct Current Stimulation—A Pilot Controlled, Randomized, Double-Blind Crossover Trial
Source: Biomedicines. 2024 Sep 2;12(9):1998. doi: 10.3390/biomedicines12091998 (PMC11428280; doi:10.3390/biomedicines12091998)
Supplement: Supplementary file 1 [file biomedicines-12-01998-s001.zip › biomedicines-3132768-Table S1.pdf]

Supplementary Material Table S1

Table 2 shows the absolute values (ms2) of LF, HF and LF/HF of participants with right and left lesions after receiving active and sham tDCS on T3 and T4, in the pre and post intervention conditions for active and sham stimulation.

	**Right Injury (N=10)**								**Left Injury (N=10)**							
	**tDCS on T3**				**tDCS on T4**				**tDCS on T3**				**tDCS on T4**			
Variables	Pre atDCS	Post atDCS	Pre sham	Post sham	Pre atDCS	Post atDCS	Pre sham	Post sham	Pre atDCS	Post atDCS	Pre sham	Post sham	Pre atDCS	Post atDCS	Pre sham	Post sham
HF (ms^2)	29,78 (18,78-40,78)	31,10 (20,10-42,10)	24,37 (13,37-35,37)	36,54 (25,54-47,54)	23,27 (12,27-34,27)	31,28 (20,28-42,28)	26,57 (15,57-37,57)	33,16 (22,16-44,16)	33,61 (22,61-44,61)	31,41 (20,41-42,41)	37,83 (26,83-48,83)	31,41 (20,41-42,41)	34,25 (23,25-45,25)	31,74 (20,74-42,74)	37,06 (26,06-48,06)	35,14 (24,14-46,14)
LF (ms^2)	70,22 (59,89-80,55)	68,9 (58,57-79,23)	75,63 (65,30-85,96)	62,93 (52,60-73,26)	76,73 (66,40-87,06)	68,72 (58,39-79,05)	73,43 (63,10-83,76)	66,84 (56,51-77,16)	66,39 (56,06-76,72)	67,13 (56,80-77,46)	62,17 (51,84-72,50)	67,13 (56,80-77,46)	65,75 (55,42-76,08)	68,26 (57,93-78,59)	62,94 (52,61-73,27)	64,86 (54,53-75,19)
HF/LF	3,25 (1,69-4,81)	3,50 (1,95-5,07)	4,57 (3,00-6,13)	2,35 (0,79-3,91)	4,41 (2,85-5,97)	4,76 (3,20-6,32)	4,66 (3,1-6,22)	2,80 (1,24-4,36)	3,02 (1,46-4,58)	3,03 (1,47-4,59)	1,93 (0,37-3,49)	3,03 (1,47-4,59)	3,12 (1,56-4,68)	3,55 (2,0-5,11)	2,89 (1,34-4,46)	1,99 (0,43-3,55)

Legend: HF = Hight Frequency, LF= Low Frequency, LF/HF: sympathovagal balance.

It is observed that individuals with lesions in both the right and left hemispheres presented a predominance of sympathetic modulation observed by the LF band (absolute values) in comparison with the parasympathetic HF action (absolute values) confirmed by the sympathovagal balance (LF/HF) >1. The results after stimulation are similar to the results in normalized units.
