# Supplementary figures and images for: The Impact of Stimulation Parameters on Cardiovascular Outcomes in Chronic Stroke Patients Following Transcranial Direct Current Stimulation—A Pilot Controlled, Randomized, Double-Blind Crossover Trial
Source: Biomedicines. 2024 Sep 2;12(9):1998. doi: 10.3390/biomedicines12091998 (PMC11428280; doi:10.3390/biomedicines12091998)

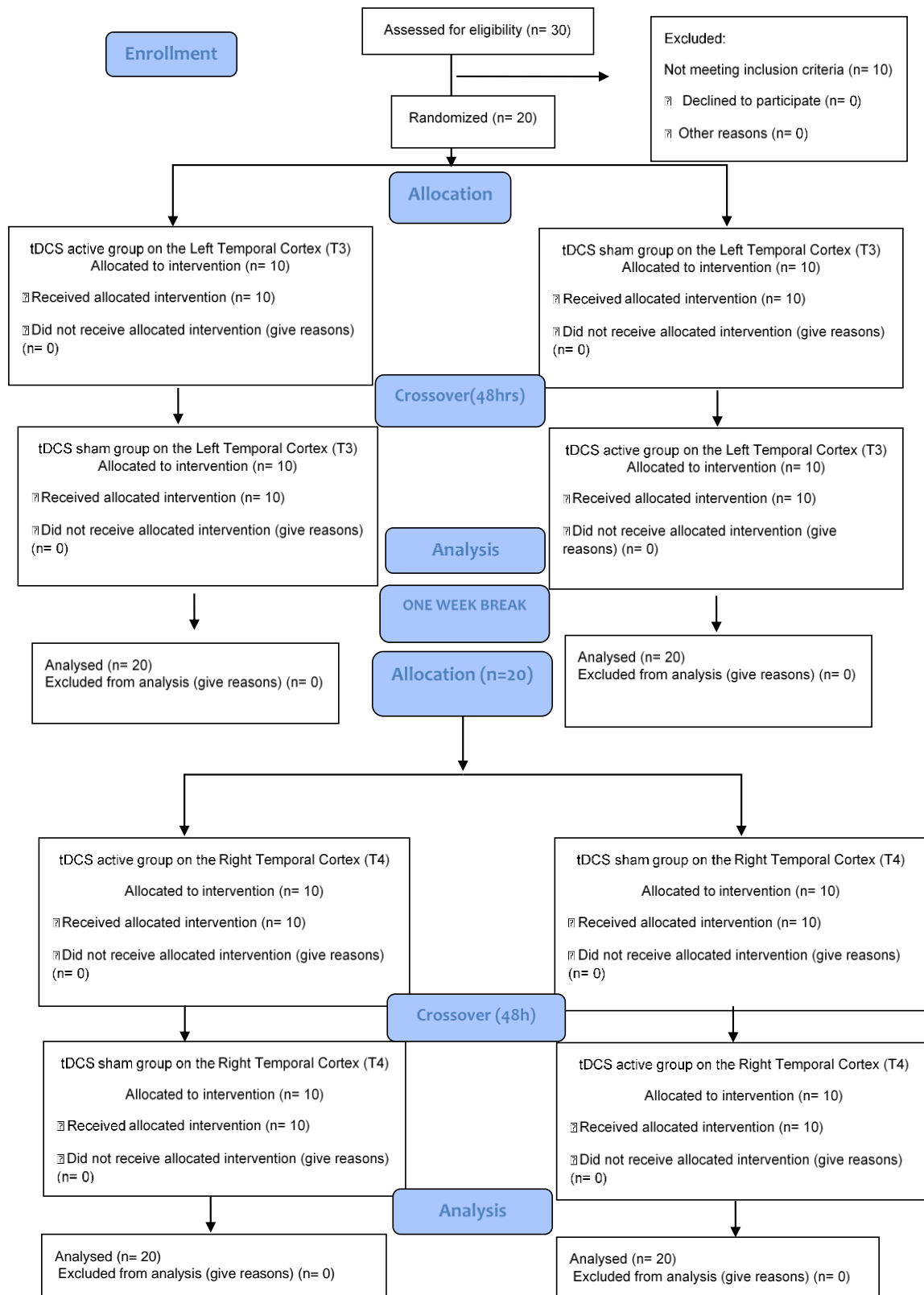

Legend: Supplementary material Figure S1 - Consort flowchart.

Supplement: Supplementary file 1 [file biomedicines-12-01998-s001.zip › biomedicines-3132768-Figure S1.pdf]
